# Supplementary figures and images for: Semantic priming and schizotypal personality: reassessing the link between thought disorder and enhanced spreading of semantic activation
Source: PeerJ. 2020 Jul 30;8:e9511. doi: 10.7717/peerj.9511 (PMC7396150; doi:10.7717/peerj.9511)

Posterior distributions  
with means and 95% intervals

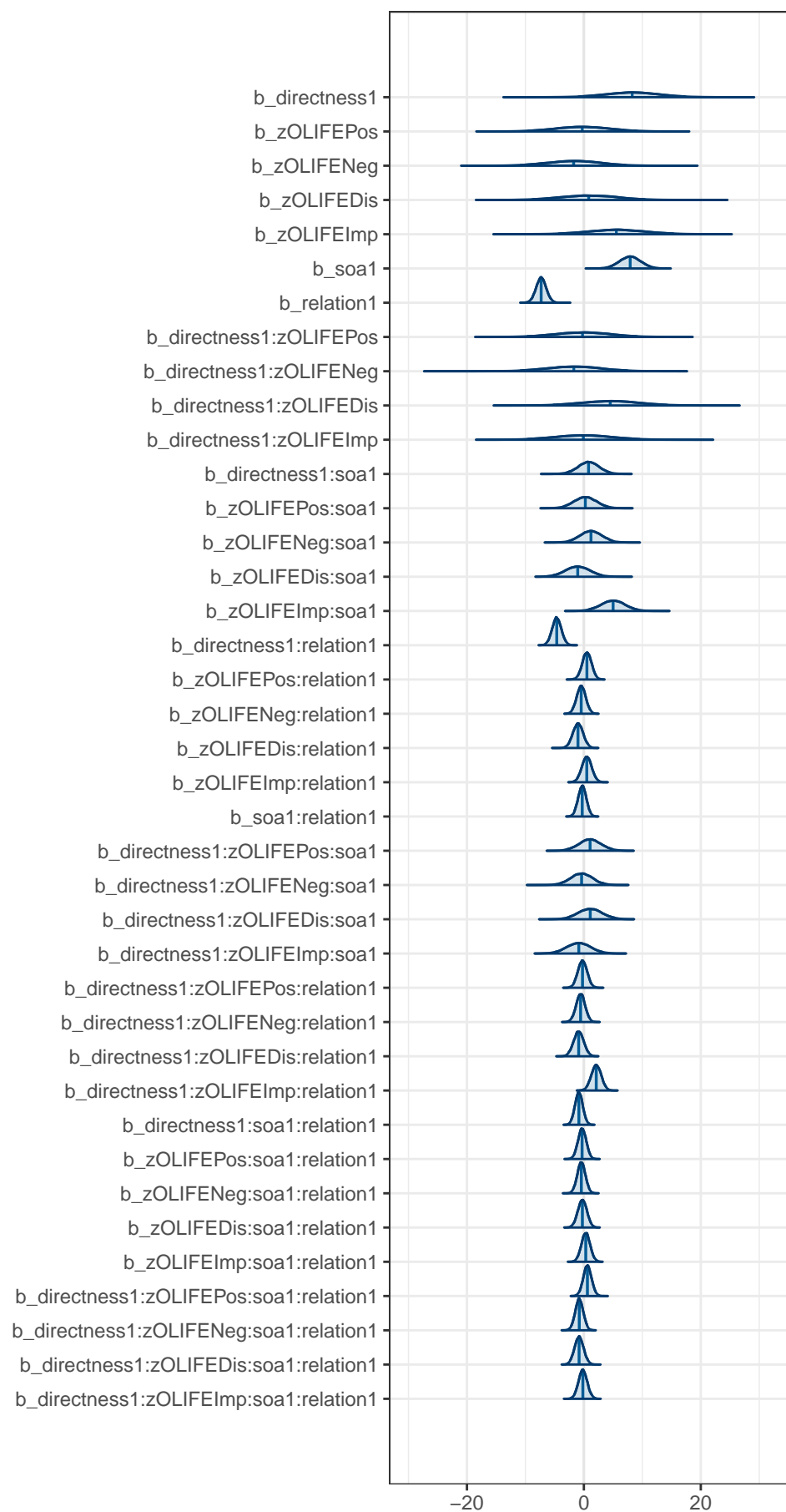

Supplement: Supplemental Information 1 [file peerj-08-9511-s001.pdf]

Directness x relatedness

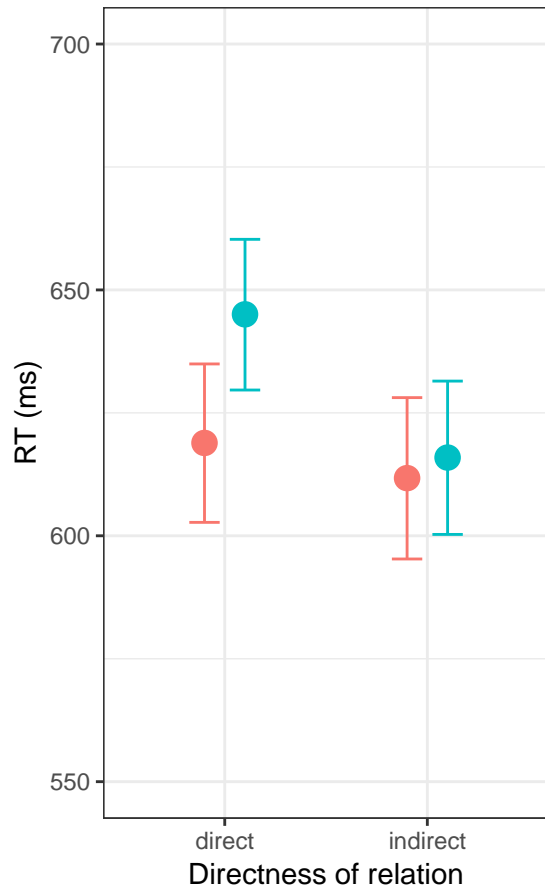

zOLIFEDis x directness

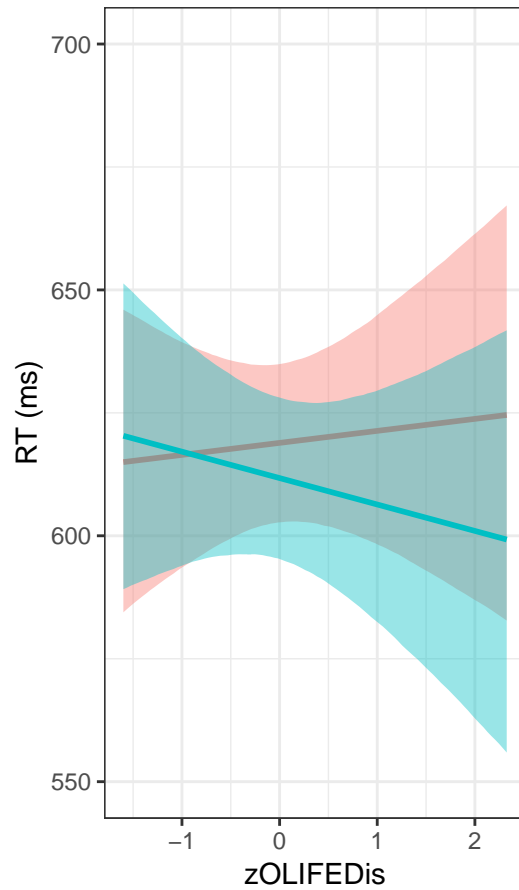

zOLIFEImp x soa

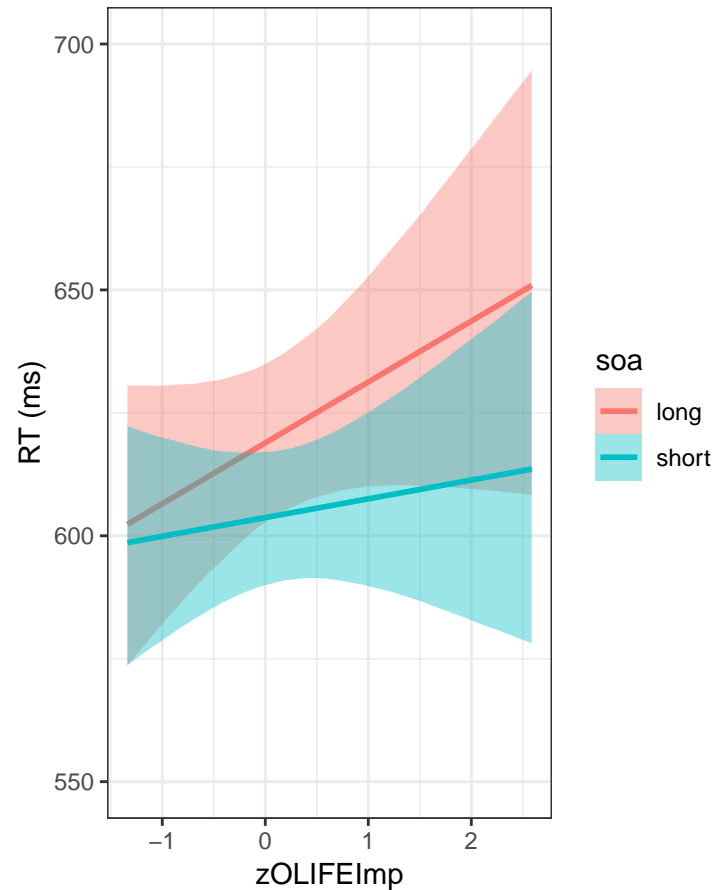

Supplement: Supplemental Information 2 [file peerj-08-9511-s002.pdf]

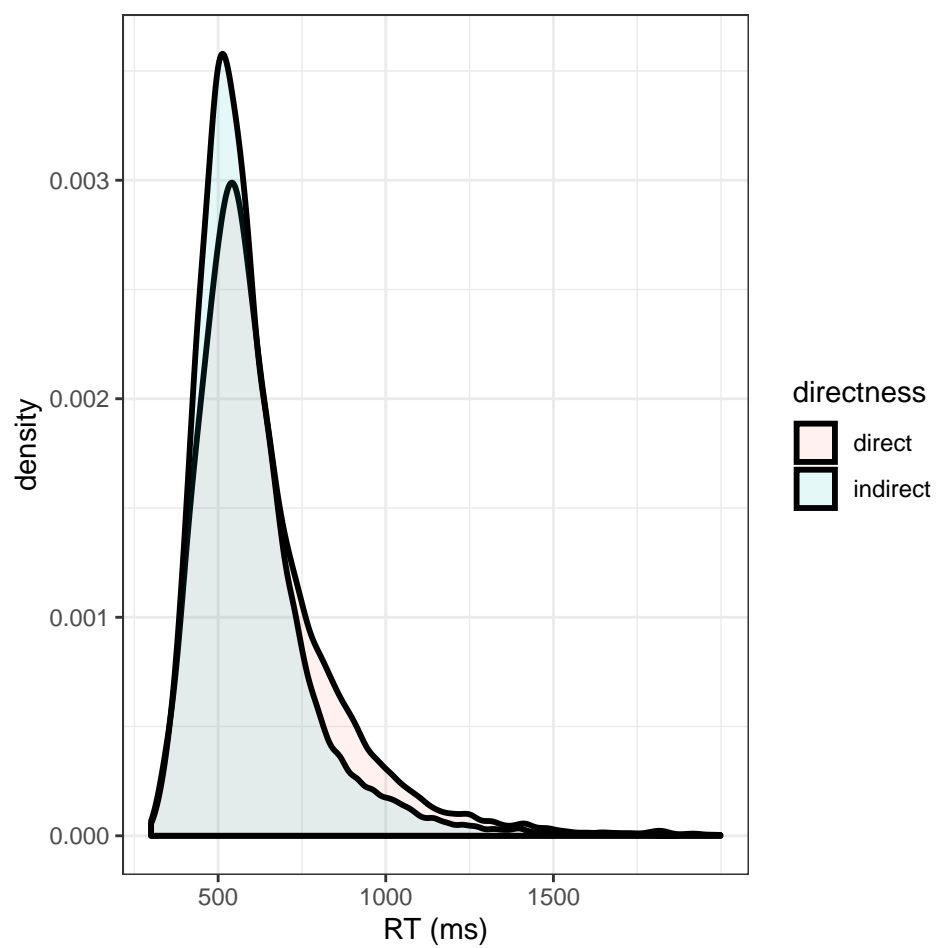

Supplement: Supplemental Information 3 [file peerj-08-9511-s003.pdf]

ExGaussian SPQ

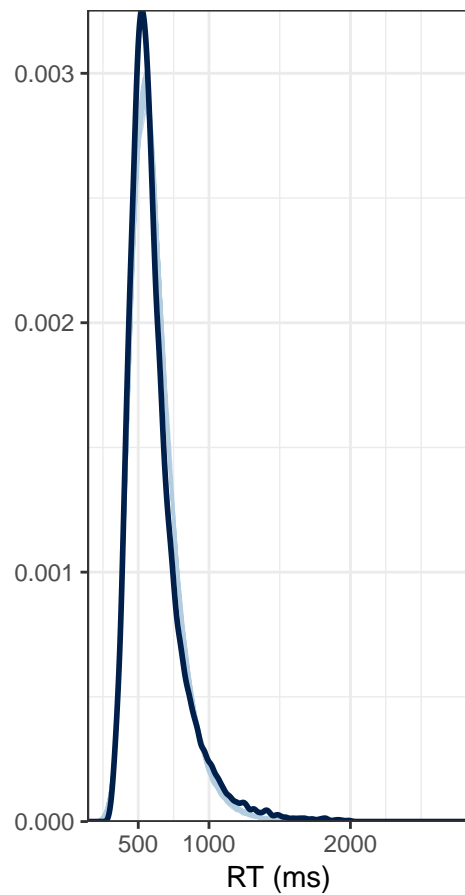

ExGaussian OLIFE

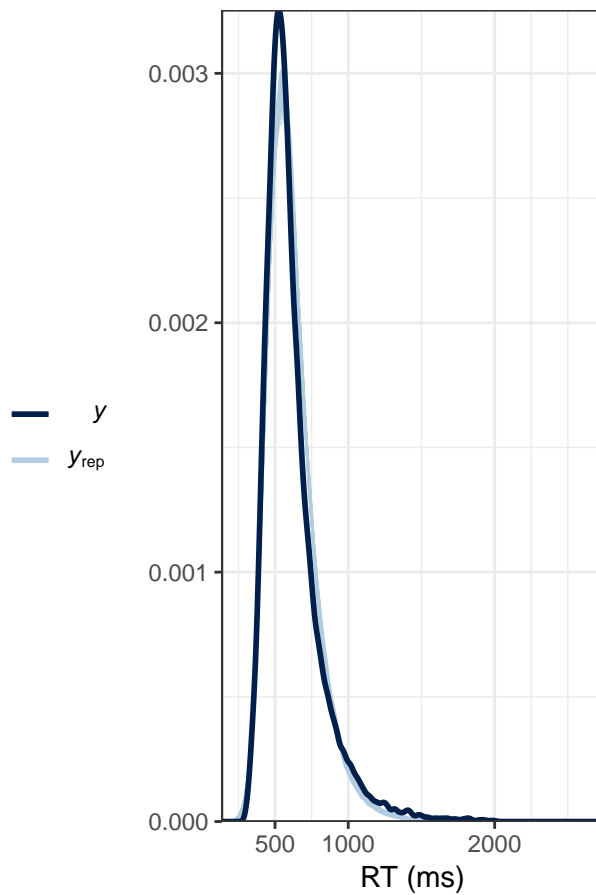

Gaussian SPQ

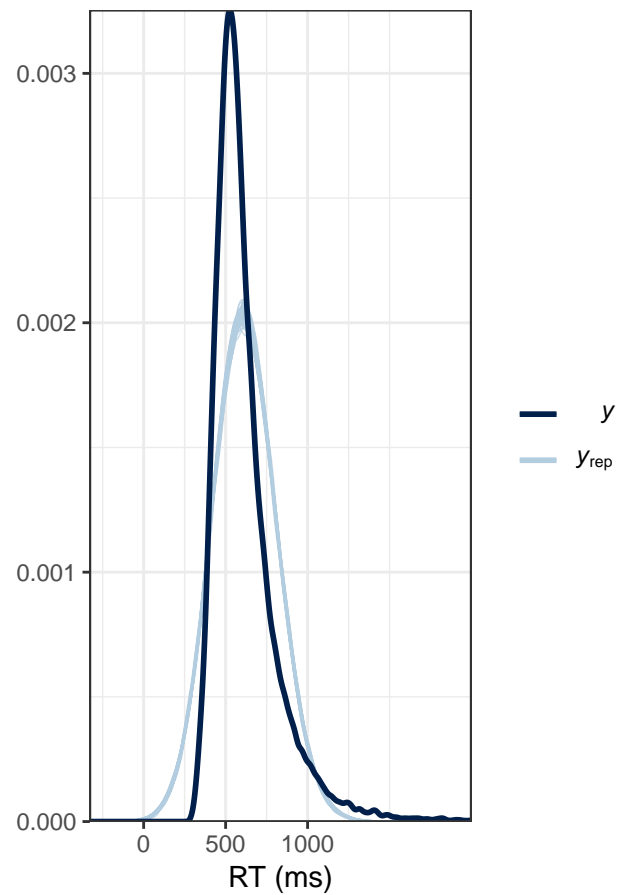

Supplement: Supplemental Information 4 [file peerj-08-9511-s004.pdf]

(a.) Estimated effect of SPQDis

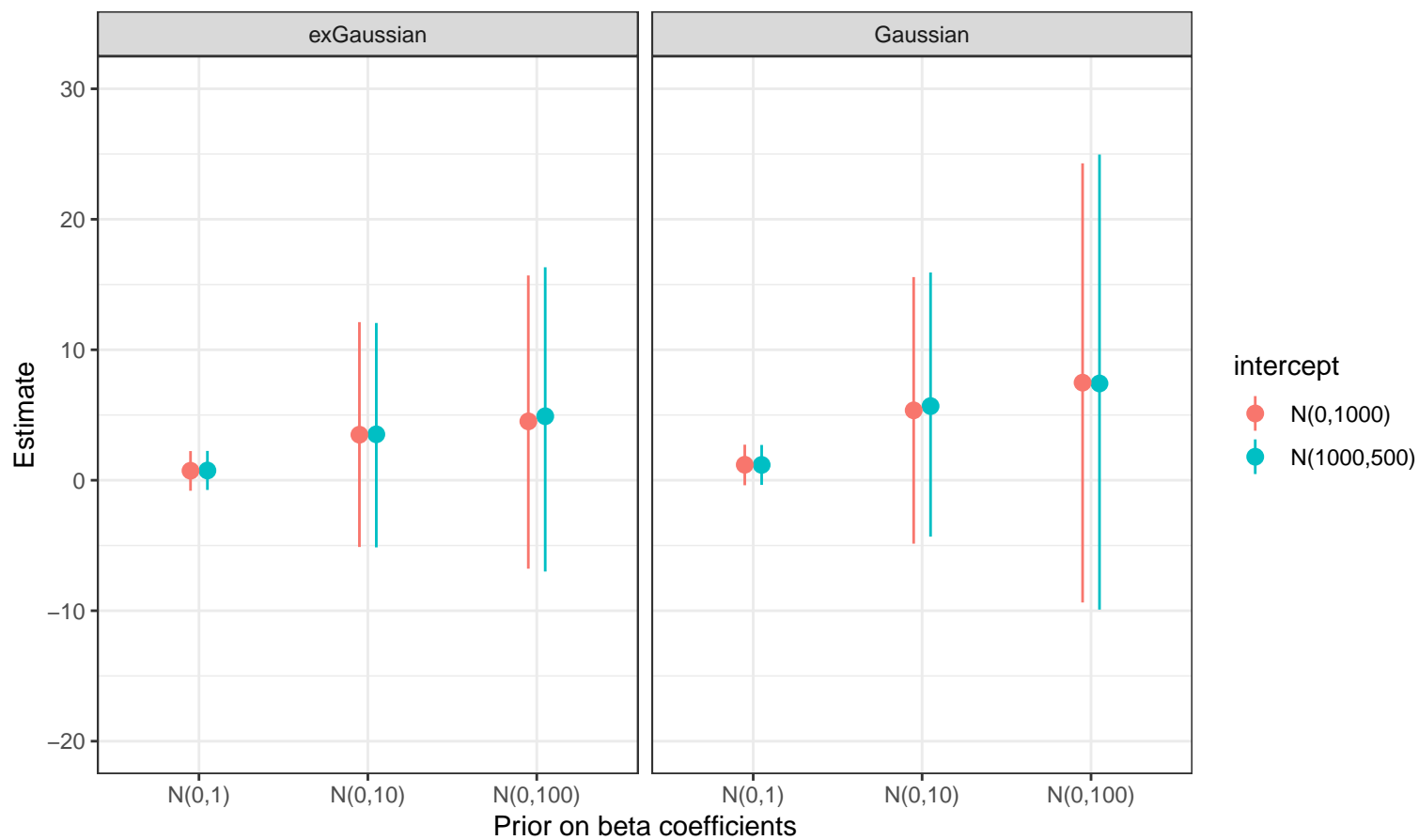

(b.) Estimated effect of OLIFEDis

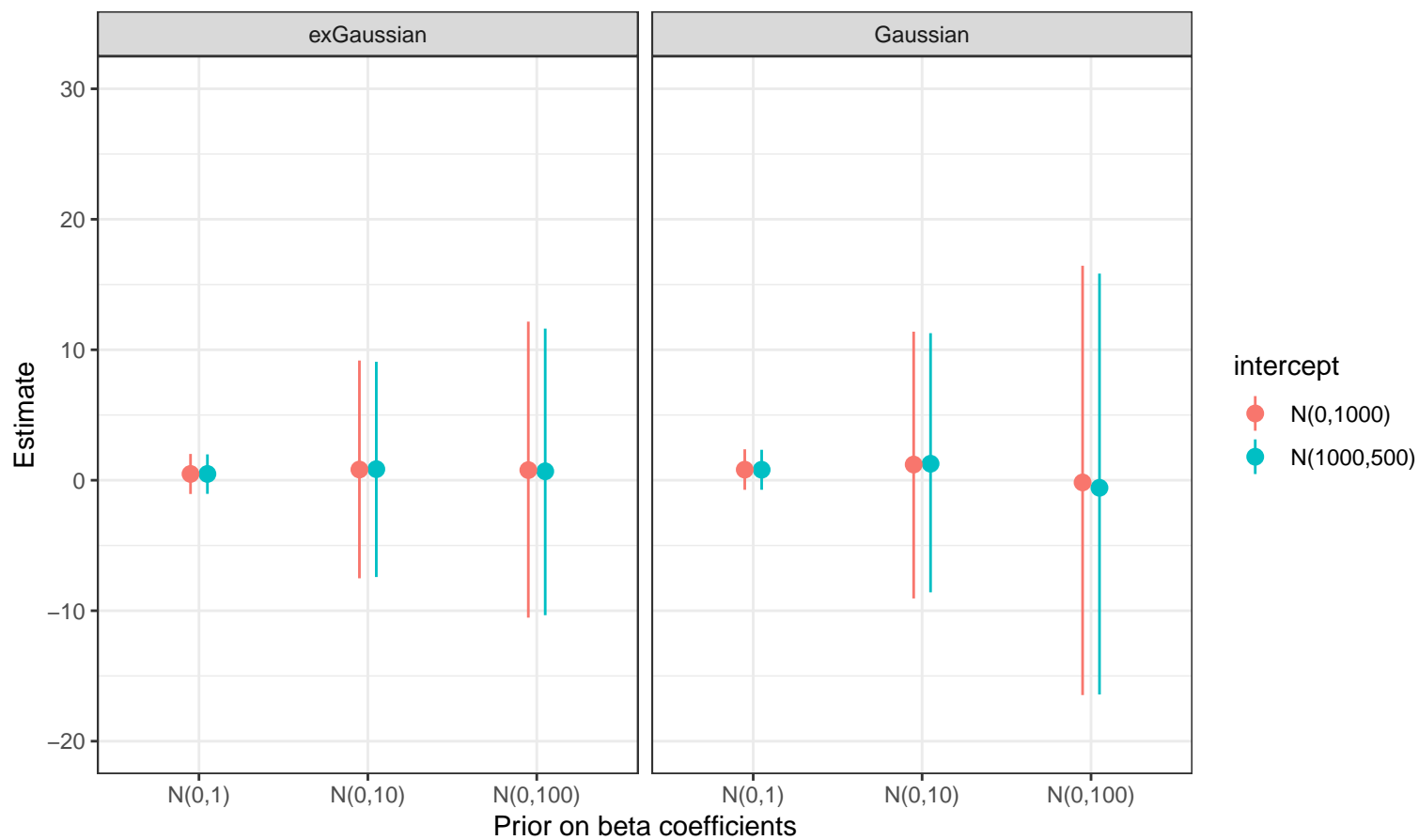

Supplement: Supplemental Information 9 [file peerj-08-9511-s009.pdf]
