# Supplementary material for: Semantic priming and schizotypal personality: reassessing the link between thought disorder and enhanced spreading of semantic activation
Source: PeerJ. 2020 Jul 30;8:e9511. doi: 10.7717/peerj.9511 (PMC7396150; doi:10.7717/peerj.9511)
Supplement: Supplemental Information 5 [file peerj-08-9511-s005.pdf]

Semantic priming and schizotypal personality: reassessing the link between thought disorder and enhanced spreading of semantic activation

Javier Rodríguez-Ferreiro[1,2], Mari Aguilera[1], Robert Davies[3]

[1] Grup de Recerca en Cognició i Llenguatge, Departament de Cognició, Desenvolupament i Psicologia de l'Educació, Universitat de Barcelona, Barcelona, Spain

[2] Institut de Neurociències, Universitat de Barcelona, Barcelona, Spain

[3] University of Lancaster, United Kingdom

Corresponding Author:
Javier Rodríguez-Ferreiro
Campus Mundet, psg de la Vall d'Hebron, 171, Barcelona, 08035, Spain
Email address: rodriguezferreiro@ub.edu

Abstract

The term schizotypy refers to a group of stable personality traits with attributes similar to symptoms of schizophrenia, usually classified in terms of positive, negative or cognitive disorganization symptoms. The observation of increased spreading of semantic activation in individuals with schizotypal traits has led to the hypothesis that thought disorder, one of the characteristics of cognitive disorganization, stems from semantic disturbances. Nevertheless, it is still not clear under which specific circumstances (i.e., automatic or controlled processing, direct or indirect semantic relation) schizotypy affects semantic priming or whether it does affect it at all. We conducted two semantic priming studies with volunteers varying in schizotypy, one with directly related prime-target pairs and another with indirectly related pairs. Our participants completed a lexical decision task with related and unrelated pairs presented at short (250 ms) and long (750 ms) stimulus onset asynchronies (SOAs). Then, they responded to the brief versions of the Schizotypal Personality Questionnaire and the Oxford-Liverpool Inventory of Feelings and Experiences, both of which include measures of cognitive disorganization. Bayesian mixed-effects models indicated expected effects of SOA and semantic relatedness, as well as an interaction between relatedness and directness (greater priming effects for directly related pairs). Even though our analyses demonstrated good sensitivity, we observed no influence of cognitive disorganization over semantic priming. Our study provides no compelling evidence that schizotypal symptoms, specifically those associated with the cognitive disorganization dimension, are rooted in an increased spreading of semantic activation in priming tasks.

Explanation of analysis approach

The use of mixed-effects models to estimate potential effects on lexical decision response latencies is warranted by the design of the study. Because we recorded multiple observations for each participant and each stimulus, and because all participants were presented with the same stimuli, we cannot assume that observed responses were independent (Baayen, Davidson, & Bates, 2008). This ruled out the use of linear models (ANOVA or regression analyses) which assume observations are independent. Moreover, because response data can be grouped by sampled participant or by sampled stimulus (prime or target), we must take into account the ways in which response latencies may have varied according to random differences between participants or between stimuli in average reaction time (random intercepts), when controlling for experimental effects, as well as the ways in which the impact of experimental factors may have varied according to random differences (random slopes) between participants or between stimuli. Taking these random effects into account in an analysis has been demonstrated to ensure more accurate estimation of the effects of interest, here, the effects of priming or SOA conditions (McElreath, 2018). In all analyses, models were fitted with maximal random effects structures (Barr, Levy, Scheepers, & Tily, 2013; Matuschek, Kliegl, Vasishth, Baayen, & Bates, 2017), corresponding to critical interaction effects (see the explanation following), to control for the risk of false positive (Type I) or negative (Type II) errors.

In our analyses, we used the *brms* library (Bayesian regression models using ‘Stan’; Bürkner, 2017, 2019; Carpenter et al., 2017) to fit Bayesian mixed-effects models. This is, in part, because Bayesian models have been found to converge essentially irrespective of model complexity whereas frequentist models may sometimes fail to converge (Eager & Roy, 2017; Matuschek et al., 2017) in situations where random effects structures are complex relative to available data. Bayesian models virtually always converge to accurate values of the parameters and produce accurate values of credible intervals for any sample (Liddell & Kruschke, 2018). It is also, in part, because Bayesian methods permit flexibility, not readily afforded under alternative approaches, to assume realistic models for the data generating processes hypothesized to underlie the production of observed responses (Martin & Williams, 2017; Nicenboim & Vasishth, 2016). In the current study, we were able to assume that response latencies were compatible with an ex-Gaussian likelihood function. The ex-Gaussian has been shown to furnish an accurate

representation of the skewed distribution typical of reaction time data (Matzke & Wagenmakers, 2009; van Zandt, 2000). As will be seen, posterior predictive checks demonstrated that this assumption enabled us to fit models that effectively captured the distribution of the reaction times observed in our study.

In general, Bayesian models are scientifically advantageous because they yield accurate representations of the posterior distribution (see e.g., Nicenboim & Vasishth, 2016; Vasishth et al., 2018, for tutorial introductions). For each parameter (including each fixed and random effect), we assumed that coefficient estimates may vary in sign and magnitude. Bayesian models yield a posterior probability distribution representing the differing probabilities of each potential value of an effect, given the observed evidence and prior expectations about likely effects. This means that, for each effect, we are able to report the most probable value of the estimate for the effect, while the spread of the posterior distribution directly indicates our uncertainty about the effect estimate. We report credible intervals (CrI) to summarize that uncertainty. In line with intuition, credibility intervals indicate the range within which we can suppose with a certain probability that the "true value" of a parameter lies given the data and the model.

The specification of model parameters reflected the features of our study design and the attributes of data collection in the sub-studies. We explain, in the following, how design or data collection features mapped to model specification choices. In particular, critical to the specification of random effects structures, we outline how experimental variables were manipulated or varied with respect to sample grouping variables (i.e., within- or between-participants or stimuli). We checked how our assumptions might potentially modulate the effects estimates in sensitivity analyses presented in supplementary materials (see Supplemental Article S2).

We recorded the latency of responses to word or pseudoword target stimuli in a lexical decision task. Each target was presented following a word prime stimulus, and our analysis focused on the latency of responses to word targets; we excluded responses to pseudowords. In each trial, the response interval lasted for 2000ms from target stimulus offset so that we only recorded lexical decisions if response onset had been less than 2000ms. We excluded observations corresponding

to latencies faster than 300ms, non-responses within the 2000ms interval, and target classification errors. Given the skew typical of response latencies in word recognition tasks, we assumed *a priori* that response latencies would be adequately described by an Ex-Gaussian function (see the discussion following), and that response latencies would be distributed in the range 300-2000ms (we checked the impact of varying this assumption, Supplemental Article S2).

The study design required the manipulation of prime-target relatedness, the directness of the prime-target relation, and prime-target stimulus onset asynchrony, in addition to the observation of participants' scores on the SPQ-B and sO-LIFE measures of variation in schizotypy dimensions. We manipulated the directness of the relation between prime and target in two separate sub-studies. As different groups of participants were recruited to different sub-studies, this means that the directness of prime-target relatedness was manipulated between-participants. The first and second sub-studies were identical in the composition of the prime stimulus set but differed in the target stimulus set so that directness was manipulated within-primes but between-targets. All participants saw each prime and target under both SOA and both relatedness so that SOA and relatedness were manipulated within-participants, within-primes and within-targets.

We fitted models that took into account the features of study design and data collection just outlined. We sum-coded the effects of the categorical variables: prime-target relatedness; SOA; and directness of prime-target relatedness. We standardized participants' scores on the schizotypy dimensions. We fitted separate models including the effects of participant variation on each dimension of one set of schizotypy scales (sO-LIFE or SPQ-B) only. Models were structured to estimate effects of these variables as well as the effects of all interactions up to and including the potential four-way interaction between the effects of directness, schizotypy dimension, SOA and relatedness. We can express this as:

RT ~ directness x schizotypy dimensions x SOA x relatedness

where the four-way interaction stands for all effects of each variable as well as all lower-order two-way interactions (including, e.g., interactions between directness and relatedness, or between schizotypal disorganization and relatedness) and all three-way interactions (including

interactions between SOA, disorganization and relatedness). While the distinction between fixed and random effects is inconsistent (Gelman & Hill, 2007), and arguably does not have force in Bayesian analyes (Nicenboim & Vasishth, 2016), we continue to use it because of its wide adoption in presentations of mixed-effects models in the literature (Meteyard & Davies, 2020). In these terms, we fitted models that included parameters corresponding to random effects associated with:

(1.) unexplained differences between sampled participants in intercepts (random intercepts) and in the within-participant effects of SOA, relatedness and the SOA x relatedness interaction (random slopes); as well as correlations between random intercepts and random slopes;

(2.) unexplained differences between sampled primes or targets in intercepts (random intercepts) and in the within-stimulus effects of participants' variation in schizotypy dimensions, and in the effects of SOA, relatedness and the SOA x relatedness interaction (random slopes); as well as differences in the within-prime effect of directedness; along with correlations between random intercepts and random slopes.

Bayesian models require the specification of prior probability distributions, in addition to the likelihood, and the fixed and random effects structure. In the present article, we report the posterior distributions of parameter estimates yielded by models assuming *weakly informative* priors for fixed effects coefficients or random effects variances: Gaussian (normal) probability distributions centered on a mean of zero with a standard deviation of 10 ($\beta \sim Normal(0, 10); SD \sim Normal(0, 10)$). This assumption of priors expresses the belief that the parameter values would lie between -20 and $+20$ with 95% probability. This range results from the fact that 95% of the probability in a Normal distribution lies within the interval $\mu \pm 2\sigma$ (see, e.g., Vasishth et al., 2018, for a discussion). However, we acknowledge that other researchers would regard alternate prior distributions as more appropriate. Therefore, we fitted a series of models with the same fixed and random effects structures but varying prior probability distributions. We report our observations on the variation in parameter estimates, in association with variation in priors, in the supplementary report on sensitivity analyses (Supplemental Article S2). In all models, we assumed the LKJ(2) prior for the correlations between random effects because it assumes that extreme values (i.e., approaching $r \pm 1$) are implausible (Vasishth et al., 2018). We also assumed that because the response interval was limited to

2000ms and, based on our experience of observing word recognition latencies, it was reasonable to suppose a prior of $\beta_0 \sim Normal(1000, 500)$ for intercepts, that is, supposing that the intercept would lie between 0-2000ms (i.e., $\mu \pm 2\sigma$) with 95% probability. (Our sensitivity analyses examined, also, the impact of this choice compared to at least one alternate, Supplemental Article S2.)

We report the estimates derived from the fitted models in the main manuscript. We describe, here, however, the distribution of observed response latencies (Supplemental Fig. S3) because the form of the distribution relates to the reasonableness of our assumption of an ex-Gaussian likelihood function in modeling the lexical decisions recorded in our sub-studies. Fig. S3 shows that the distributions of observed RTs for each sub-study have similar forms, with peaks between 500-700ms, with variation in the shorter RTs appearing to conform to a Gaussian (normal) distribution, and longer RTs presenting a marked skew appearing to conform to an exponential modification of the Gaussian. The observed RT distribution is typical for a word recognition task and it is congruent with the assumption, implemented in our models, that reaction times are generated by a process that can be described by defining an ex-Gaussian likelihood function. Our intention, in choosing the ex-Gaussian, was simply to arrive at a good description of observed latencies but alternate likelihood functions are available, including those that correspond to the lognormal (e.g. Nicenboim & Vasishth, 2016), shifted lognormal (Vasishth et al., 2018), or skew normal (Martin & Williams, 2017). We share our data and analysis code to enable readers to examine the impact on parameter estimates of varying the likelihood function; we report our own examination of this question in the sensitivity analyses.

The full dataset and code for the analyses are available at OSF: https://osf.io/j29fn/.

References

Baayen, R. H., Davidson, D. J., & Bates, D. M. (2008). Mixed-effects modeling with crossed random effects for subjects and items. *Journal of Memory and Language, 59*, 390–412.

Barr, D. J., Levy, R., Scheepers, C., & Tily, H. J. (2013). Random effects structure for confirmatory hypothesis testing : Keep it maximal. *Journal of Memory and Language*, *68*, 255–278. https://doi.org/10.1016/j.jml.2012.11.001

Bürkner, P. (2017). brms: An R Package for Bayesian Multilevel Models Using Stan. *Journal of Statistical Software*, *80*, 1–28.

Bürkner, P. (2019). *brms: Bayesian Regression Models using "Stan."* Retrieved from https://cran.r-project.org/web/packages/brms/brms.pdf

Carpenter, B., Gelman, A., Hoffman, M., Lee, D., Goodrich, B., Betancourt, M., … Ridell, A. (2017). Stan: A Probabilistic Programming Language. *Journal of Statistical Software*, *76*, 1–32.

Eager, C., & Roy, J. (2017). Mixed effects models are sometimes terrible. *ArXiv Preprint ArXiv:1701.04858.*

Gelman, A., & Hill, J. (2007). *Data analysis using regression and hierarchical/multilevel models*. New York, NY: Cambridge University Press.

Liddell, T. M., & Kruschke, J. K. (2018). Analyzing ordinal data with metric models: What could possibly go wrong? *Journal of Experimental Social Psychology*, *79*, 328–348.

Martin, S. R., & Williams, D. R. (2017). Outgrowing the Procrustean Bed of Normality: The Utility of Bayesian Modeling for Asymmetrical Data Analysis. *PsyArXiv*.

Matuschek, H., Kliegl, R., Vasishth, S., Baayen, H., & Bates, D. (2017). Balancing Type I error and power in linear mixed models. *Journal of Memory and Language*, *94*, 305–315.

Matzke, D., & Wagenmakers, E. J. (2009). Psychological interpretation of the ex-Gaussian and shifted Wald parameters: A diffusion model analysis. *Psychonomic Bulletin & Review*, *16*, 798-817.

McElreath, R. (2018). *Statistical rethinking: A bayesian course with examples in R and Stan*. Chapman & Hall/CRC Press.

Meteyard, L., & Davies, R. A. (2020). Best practice guidance for linear mixed-effects models in psychological science. *Journal of Memory and Language*, *112*, 104092.

Nicenboim, B., & Vasishth, S. (2016). Statistical methods for linguistic research: Foundational Ideas—Part II. *Language and Linguistics Compass*, *10*, 591-613.

Van Zandt, T. (2000). How to fit a response time distribution. *Psychonomic Bulletin & Review*, *7*, 424-465.

Vasishth, S., Nicenboim, B., Beckman, M. E., Li, F., & Kong, E. J. (2018). Bayesian data analysis in the phonetic sciences: A tutorial introduction. *Journal of Phonetics*, *71*, 147-161.
