# Supplementary material for: Semantic priming and schizotypal personality: reassessing the link between thought disorder and enhanced spreading of semantic activation
Source: PeerJ. 2020 Jul 30;8:e9511. doi: 10.7717/peerj.9511 (PMC7396150; doi:10.7717/peerj.9511)
Supplement: Supplemental Information 6 [file peerj-08-9511-s006.pdf]

Semantic priming and schizotypal personality: reassessing the link between thought disorder and enhanced spreading of semantic activation

Javier Rodríguez-Ferreiro[1,2], Mari Aguilera[1], Robert Davies[3]

[1] Grup de Recerca en Cognició i Llenguatge, Departament de Cognició, Desenvolupament i Psicologia de l'Educació, Universitat de Barcelona, Barcelona, Spain

[2] Institut de Neurociències, Universitat de Barcelona, Barcelona, Spain

[3] University of Lancaster, United Kingdom

Corresponding Author:

Javier Rodríguez-Ferreiro

Campus Mundet, psg de la Vall d'Hebron, 171, Barcelona, 08035, Spain

Email address: rodriguezferreiro@ub.edu

Abstract

The term schizotypy refers to a group of stable personality traits with attributes similar to symptoms of schizophrenia, usually classified in terms of positive, negative or cognitive disorganization symptoms. The observation of increased spreading of semantic activation in individuals with schizotypal traits has led to the hypothesis that thought disorder, one of the characteristics of cognitive disorganization, stems from semantic disturbances. Nevertheless, it is still not clear under which specific circumstances (i.e., automatic or controlled processing, direct or indirect semantic relation) schizotypy affects semantic priming or whether it does affect it at all. We conducted two semantic priming studies with volunteers varying in schizotypy, one with directly related prime-target pairs and another with indirectly related pairs. Our participants completed a lexical decision task with related and unrelated pairs presented at short (250 ms) and long (750 ms) stimulus onset asynchronies (SOAs). Then, they responded to the brief versions of the Schizotypal Personality Questionnaire and the Oxford-Liverpool Inventory of Feelings and Experiences, both of which include measures of cognitive disorganization. Bayesian mixed-effects models indicated expected effects of SOA and semantic relatedness, as well as an interaction between relatedness and directness (greater priming effects for directly related pairs). Even though our analyses demonstrated good sensitivity, we observed no influence of cognitive disorganization over semantic priming. Our study provides no compelling evidence that schizotypal symptoms, specifically those associated with the cognitive disorganization dimension, are rooted in an increased spreading of semantic activation in priming tasks.

Sensitivity analyses

Our models were specified according to a number of assumptions. We motivated our assumptions by reasoning that proceeded from the implications of the study design, and the features of the data collection method, to develop a statistical account of the cognitive processes that could generate the responses recorded in the lexical decision task. Our reasoning is explained in the main article but we acknowledge that other researchers might make different decisions about the most appropriate analysis than those we adopted, in addressing the same research questions (Goodman, Fanelli, & Ioannidis, 2016) or, indeed, in examining the same data (cf. Silberzahn et al., 2018). We share our data and analysis code to facilitate *methods reproducibility* (Goodman et al., 2016), enabling other researchers to reproduce the results we present in our report. In sharing our data and code, in addition, we seek to enable other researchers to address the same questions, with the same data, supposing different analytic choices. This is because we think reasonable people may differ, on decisions pertaining to an analysis, in response to the study design or data collection method we have described (as demonstrated e.g., by Silberzahn et al., 2018), and we embrace this potential diversity as a means to establish the generalizability of empirical findings. We follow Gelman and Hennig (2016), therefore, by seeking to account for our assumptions or decisions in the main article and, here, by conducting an analysis of the stability of estimates given potential variation in approach. For our purposes (of course, other researchers may differ even in this), our sensitivity analyses were done to examine how effects estimates might vary given different decisions about the likelihood function, or about the Bayesian priors (see, also, Depaoli and van de Schoot, 2017). (We offer readers one caveat: one reason why we did not base our results on frequentist models of the data, using, for example, the *lme4* library (Bates et al., 2019) is that we found that models with similar random effects specifications did not converge; see Supplemental Article S3.)

Likelihood functions and posterior predictive checks

If a model is a good fit then we should be able to use it to generate data similar to the data we observed (Gabry, 2017; Gabry, Simpson, Vehtari, Betancourt, & Gelman, 2019). To generate the data used for such posterior predictive checks (PPC), we simulate them from the posterior predictive distribution. This is the distribution of the outcome variable implied by a model after using the observed data to update our beliefs about unknown model parameters.

We explained that the reaction time distributions for tasks like lexical decision are typically observed to be skewed towards longer latencies (see Supplemental Article S1) and this form has been argued to be well described as an ex-Gaussian distribution (e.g., Matzke & Wagenmakers, 2009; van Zandt, 2000). (The density plots depicting the distribution of observed reaction times in our study can be seen in Supplemental Fig. S3.) We opted, therefore, to assume an ex-Gaussian likelihood function at the core of the Bayesian models fitted to the data in our study. Assuming an ex-Gaussian likelihood meant that we did not have to suppose that observed latencies had a normal distribution despite the skew clearly indicated in Figure S3 but *we could have* assumed a Gaussian likelihood. A comparison of the PPC plots in Figure S4 shows that, in fact, with models that assumed an ex-Gaussian likelihood (plots in left and center panels), there was an excellent fit for the observed latencies. Figure S4 presents overlaid density plots, showing for each model the distribution of observed latencies (dark blue, labelled "y") overlaid with outcomes simulated from the posterior predictive distribution (light blue, labelled "y_rep"), given the model.

[Figure S4 about here]

Figure S4. Posterior predictive check plots for key model variants

It is in striking contrast that, if one were to assume a Gaussian likelihood function, while the MCMC-sampling was efficient and the parameter estimates were similar to those we report, the posterior predictive check showed a marked discrepancy between the distribution of model predicted response latencies and the distributed of observed latencies (Figure S4, right-most panel).

Variation in priors and variation in estimates

In analyzing the study data, we were aware that there would be reasonable latitude about the assumption of prior probability distributions for model parameters. We opted to assume regularizing or weakly informative priors (see Nicenboim & Vasishth, 2016, for discussion) expressing the assumption that parameter estimates (e.g., the coefficients of effects) would be found to occur, with potential variation in value, according to a probability distribution identified

as a Gaussian, broadly spread, probability distribution centered on zero. We assumed this because effects of variables like SOA or relatedness could conceivably be positive or negative but were unlikely to take extreme values. Beyond these assumptions, we supposed that reasonable people might differ on the most appropriate prior probability distributions for model parameters, including fixed effects coefficients or random effects variances. Therefore, we evaluated what impact our choice versus alternate choices of priors would have on the posterior distributions of parameters, conducting an analysis thereby to examine the sensitivity of estimates to the choice of priors (e.g., Depaoli & van de Schoot, 2017; Vasishth et al., 2018), in other words, to examine the stability of estimates (Gelman & Hennig, 2016).

In the main article, we report the posterior distributions of parameters found with models assuming priors for fixed effects or random effects variances centered on a mean of zero with a standard deviation of 10 ($\beta \sim Normal(0,10); SD \sim Normal(0,10)$), expressing the belief that the parameter values would lie between -20 and $+20$ with 95% probability. However, it is possible that other researchers would regard prior distributions of $\beta \sim Normal(0,1); SD \sim Normal(0,1)$ or $\beta \sim Normal(0,100); SD \sim Normal(0,100)$ as more appropriate. Therefore, we fitted a series of models with the same fixed and random effects structures but varying prior probability distributions. In half of the model variants, we fitted models assuming a prior probability distribution for intercepts of $\beta_0 \sim Normal(0,1000)$. However, we understood that this prior distribution would allow, unrealistically, for intercepts of negative RT. We also understood that because the response interval was limited to 2000ms it was more reasonable, perhaps, to suppose a prior of $\beta_0 \sim Normal(1000,500)$.

We plot the variation in estimates (the posterior distribution mean) of the effects of SOA, relatedness, directness and the directness by relatedness interaction (Figure S5). The plots indicate how parameter estimates vary with alternate choices of likelihood function (Gaussian vs. ex-Gaussian), intercept prior ($\beta_0 \sim Normal(0,1000)$ vs. $\beta_0 \sim Normal(1000,500)$), and fixed effects or random effects variance priors (SDs of 1, 10, or 100). Figure S5 presents a separate grid of plots for each effect: relatedness, directness, directness x relatedness, and SOA. In each grid, we present (on separate rows) plots showing effects estimates (coefficient values) for SPQ-B versus sO-LIFE models, and (in separate columns) plots showing effects estimates

from models assuming ex-Gaussian versus Gaussian likelihood functions. In each plot, we present point estimates (points correspond to the mean of the posterior) along with credible intervals (95% lower vs. upper bounds) for effects given variation (on the x-axis) of priors (N(0,1), N(0,10), N(0,100), where N stands for *Normal*). In each plot, also, we show the estimates that derive from models assuming different priors on intercepts, either $\beta_0 \sim Normal(0{,}1000)$ or $\beta_0 \sim Normal(1000{,}500)$.

Figure S5 shows that, for the effects about which we can have reasonably high certainty (the effects of SOA, relatedness, and the directness x relatedness interaction), variation in the spread (SD) of the prior probability distributions (N(0,1) vs. N(0,10) vs. N(0,100)) is associated with limited variation in the magnitude of effects but no variation in the direction of effects. In this comparison, with narrowly spread priors ($\beta \sim Normal(0{,}1)$), estimates for the effects of relatedness, SOA, and of the directness x relatedness interaction are smaller (the tight priors, centered on 0, pull estimates towards them more strongly) but it remains clear that the evidence shows that the effects are present in the data with the signs (positive or negative) we report irrespective of likelihood function, or prior choice, among the choices we examined. In comparison, for effects for which we have relatively weak evidence in this study (the effects of directness and of SPQDis, OLIFEDis variation, Figure S6), parameter estimates vary markedly with respect to the width of credibility intervals but not with respect to the point estimates (the values of the most probable estimates for the coefficients of the effects).

[Figures S5 and S6 about here]

Figure S5. Variation in relatedness, directness, directness x relatedness, and SOA effects estimates by model variants

Figure S6. Variation in SPQDis and OLIFEDis effects estimates by model variants

Conclusions

We conclude that the estimates derived from our models for the experimental effects of, especially, relatedness, directness, SOA, and the directness x relatedness interaction are stable across a range of model variants, fitted with alternate assumptions.

The full dataset and code for the analyses are available at OSF: https://osf.io/j29fn/.

References

Bates, D., Maechler, M., Bolker, B., et al. (2019). lme4: Linear Mixed-Effects Models using 'Eigen' and S4. R package version 1.1-21. https://cran.r-project.org/web/packages/lme4/lme4.pdf

Depaoli, S., & Van de Schoot, R. (2017). Improving transparency and replication in Bayesian statistics: The WAMBS-Checklist. *Psychological Methods*, *22*, 240-261.

Gabry, J. (2017) bayesplot: plotting for Bayesian models. *R Package Version 1.3.0.* Columbia University, New York. (Available from http://mc-stan.org/bayesplot.)

Gabry, J., Simpson, D., Vehtari, A., Betancourt, M., & Gelman, A. (2019). Visualization in Bayesian workflow. *Journal of the Royal Statistical Society: Series A (Statistics in Society), 182*, 389-402.

Gelman, A., & Hennig, C. (2017). Beyond subjective and objective in statistics. *Journal of the Royal Statistical Society: Series A (Statistics in Society)*, *180*, 967-1033.

Goodman, S. N., Fanelli, D., & Ioannidis, J. P. (2016). What does research reproducibility mean? *Science Translational Medicine*, *8*, 341-347.

Matzke, D., & Wagenmakers, E. J. (2009). Psychological interpretation of the ex-Gaussian and shifted Wald parameters: A diffusion model analysis. *Psychonomic Bulletin & Review*, *16*, 798-817.

Nicenboim, B., & Vasishth, S. (2016). Statistical methods for linguistic research: Foundational Ideas—Part II. *Language and Linguistics Compass*, *10*, 591-613.

Silberzahn, R., Uhlmann, E. L., Martin, D. P., Anselmi, P., Aust, F., Awtrey, E., ... & Carlsson, R. (2018). Many analysts, one data set: Making transparent how variations in analytic choices affect results. *Advances in Methods and Practices in Psychological Science*, *1*, 337-356.

Van Zandt, T. (2000). How to fit a response time distribution. *Psychonomic Bulletin & Review*, *7*, 424-465.

Vasishth, S., Nicenboim, B., Beckman, M. E., Li, F., & Kong, E. J. (2018). Bayesian data analysis in the phonetic sciences: A tutorial introduction. *Journal of Phonetics*, *71*, 147-161.
