# Supplementary material for: Semantic priming and schizotypal personality: reassessing the link between thought disorder and enhanced spreading of semantic activation
Source: PeerJ. 2020 Jul 30;8:e9511. doi: 10.7717/peerj.9511 (PMC7396150; doi:10.7717/peerj.9511)
Supplement: Supplemental Information 7 [file peerj-08-9511-s007.pdf]

Semantic priming and schizotypal personality: reassessing the link between thought disorder and enhanced spreading of semantic activation

Javier Rodríguez-Ferreiro[1,2], Mari Aguilera[1], Robert Davies[3]

[1] Grup de Recerca en Cognició i Llenguatge, Departament de Cognició, Desenvolupament i Psicologia de l'Educació, Universitat de Barcelona, Barcelona, Spain

[2] Institut de Neurociències, Universitat de Barcelona, Barcelona, Spain

[3] University of Lancaster, United Kingdom

Corresponding Author:
Javier Rodríguez-Ferreiro
Campus Mundet, psg de la Vall d'Hebron, 171, Barcelona, 08035, Spain
Email address: rodriguezferreiro@ub.edu

Abstract

The term schizotypy refers to a group of stable personality traits with attributes similar to symptoms of schizophrenia, usually classified in terms of positive, negative or cognitive disorganization symptoms. The observation of increased spreading of semantic activation in individuals with schizotypal traits has led to the hypothesis that thought disorder, one of the characteristics of cognitive disorganization, stems from semantic disturbances. Nevertheless, it is still not clear under which specific circumstances (i.e., automatic or controlled processing, direct or indirect semantic relation) schizotypy affects semantic priming or whether it does affect it at all. We conducted two semantic priming studies with volunteers varying in schizotypy, one with directly related prime-target pairs and another with indirectly related pairs. Our participants completed a lexical decision task with related and unrelated pairs presented at short (250 ms) and long (750 ms) stimulus onset asynchronies (SOAs). Then, they responded to the brief versions of the Schizotypal Personality Questionnaire and the Oxford-Liverpool Inventory of Feelings and Experiences, both of which include measures of cognitive disorganization. Bayesian mixed-effects models indicated expected effects of SOA and semantic relatedness, as well as an interaction between relatedness and directness (greater priming effects for directly related pairs). Even though our analyses demonstrated good sensitivity, we observed no influence of cognitive disorganization over semantic priming. Our study provides no compelling evidence that schizotypal symptoms, specifically those associated with the cognitive disorganization dimension, are rooted in an increased spreading of semantic activation in priming tasks.

Frequentist analyses

The models we report in the article were fitted using the Bayesian brms library (Burkner, 2017, 2019). We think that these models account for the observed behaviours appropriately, incorporating information about the probability distribution of likely effects (priors), and taking into account random effects due to unexplained differences between sampling units (participants, target or prime stimuli). However, an alternative approach to analyse our data would be to employ frequentist mixed-effects model-fitting functions, such as those provided in the lme4 library (Bates et al., 2019). The key difference between the lme4 and brms model fits, arguably, is that the brms models incorporate information about relatively constraining assumptions about the (e.g. Gaussian) shape of the prior probability distributions of estimated effects, while the lme4 models incorporate information about unconstrained assumptions (so-called flat priors) about the prior probability distributions of estimated effects.

To enable readers to compare the estimates from brms and lme4 model fits, we conducted a set of supplementary analyses. We enclose the analysis files:
all-lme4-wid-2_2020-02-28.R
all-lme4-wid-1_2020-02-28.R

The .R scripts are written to run with the datasets:
PrimDir-111019.csv
PrimInd-111019.csv

The models in the .R scripts are specified to run using the lme4 library (Bates et al., 2019) in R version 3.5.1 (2018-07-02; R Core Team, 2018).

The models in the .R scripts are specified with the same fixed effects as the models reported in the article (see Manuscript tables 1 and 2, and Supplemental tables 1 and 2), but we varied random effects structure to include (see ...2020-02-28.R files):

(ri.) just random intercepts;

(rs.) random intercepts and random slopes but not covariances between intercepts and slopes deviations;
(max.) all possible random intercepts and slopes.

We ran the models on the Lancaster University HEC, then downloaded the results. The model estimates are copied to:
all-lme4-wid-2_2020-02-28-results.txt
all-lme4-wid-1_2020-02-28-results.txt

A comparison of the estimates of effects for the models we report (see Supplemental tables 1 and 2) and the frequentist models we fitted shows that the effects estimates are largely comparable in the following senses.

(1.) Where we have good evidence for the presence, size and direction of an effect -- in the reported (Bayesian) brms models, where credible intervals are relatively narrow and exclude 0 -- there is a good match between the estimates for such effects in the different kinds of models. In the (frequentist) lme4 models, typically, such effects are significant ($t > 2$).

(2.) There is divergence in the numeric values of coefficient estimates but estimates remain similar i.e. within an order of magnitude, comparing brms and lme4 model estimates. There is relatively more divergence, we observe, where there is weak evidence for an effect i.e. if credible intervals are wider, and if they include 0; in the frequentist models, such effects are typically non-significant.

(3.) Where there might be what is conventionally described as near-significant or just-significant effects e.g. the effect of directness (where $t \sim 2.5$) there the Bayesian model estimate is smaller (~7ms) than the frequentist model estimate (~20ms) but we think that the frequentist model estimate is an over-estimate of the effect given the fact that frequentist models assume that very large or very small effects are equally probable (hence, assume flat prior probability distributions for effects), unlike the Bayesian models reported here which assume Gaussian (normal) prior probability distributions corresponding to potential values of effects estimates. (We checked if

variation in choices, in our models, of likelihood function or the narrowness or spread of prior distributions influenced effects estimates and found that they did not do so; the scripts specified to run the sensitivity checks are also located in Supplementary Materials.)

Critically, we found that all the models run to completion but the rs and max models were associated with convergence warnings:
> convergence code: 0
> boundary (singular) fit: see ?isSingular

How concerned should we about such warnings? The singular fit warning indicates that there is a problem, for lme4, fitting the model specified, given the data being analysed. Bates et al. (2018) comment (p.3):

“Almost unfortunately, the software does indeed converge to parameter estimates but these estimates correspond to degenerate or singular covariance matrices, in which some linear combinations of the random effects are estimated to having no variability. This corresponds to estimates of zero random-effects variance in a model with random-intercepts only or a correlation of +/- in a model with correlated random intercepts and slopes. ... In summary, the parameters representing variances and covariances are constrained in complicated ways. In overparameterized models, convergence can occur on the boundary, corresponding to models with singular variance-covariance matrices for random effects. This can have serious, adverse consequences for inference...”

We suppose that it is necessary to fit models including all hypothesised effects, and all potential associated random effects, as we explain in the manuscript. Alongside this aim, it is important that a statistical model incorporates information about the prior probability distribution of possible effects in a domain of study, included in the form of prior distribution information in Bayesian models. However, in addition, as has been widely noted (e.g., Martin & Williams, 2017), we note that more complicated model specifications can run into convergence difficulties or run in singular fits, if flat priors are assumed, as in frequentist models, while Bayesian models will practically always converge.

We are aware that convergence problems can be addressed by specifying models with simpler structures, typically, in our experience, by simplifying the random effects structure (see, for discussion, Bates et al., 2018; Meteyard & Davies, 2020). However, we were concerned about the proliferation of researcher degrees of freedom around the choices that would have to be made in order to effectively simplify the random effects for models like ours. Fitting Bayesian mixed-effects models also requires decision making -- and we checked the consequences of our choices in the sensitivity analyses -- but also permitted us to keep the analysis approach relatively simple, avoiding a process of model selection.

Conclusions

We conclude that the estimates derived from our models for the experimental effects of, especially, relatedness, directness, SOA, and the directness x relatedness interaction are stable across a range of model variants, fitted with alternate assumptions.

The full dataset and code for the analyses are available at OSF: https://osf.io/j29fn/.

References

Bates, D., Kliegl, R., Vasishth, S., & Baayen, H. (2018). Parsimonious mixed models. arXiv preprint https://arxiv.org/pdf/1506.04967.pdf.

Bates, D., Maechler, M., Bolker, B., Walker, S., Christensen, R. H. B., Singmann, H., . . . Fox, J. (2019). lme4: Linear mixed-effects models using Eigen and S4. R package version, 1.1-21. Library information retrieved from: https://cran.r-project.org/web/packages/lme4/lme4.pdf

Bürkner, P. (2017). brms: An R Package for Bayesian Multilevel Models Using Stan. *Journal of Statistical Software*, 80, 1–28.

Bürkner, P. (2019). brms: Bayesian Regression Models using "Stan." Retrieved from https://cran.r-project.org/web/packages/brms/brms.pdf

Martin, S. R., & Williams, D. R. (2017). Outgrowing the Procrustean Bed of Normality: The Utility of Bayesian Modeling for Asymmetrical Data Analysis. Retrieved from: https://osf.io/9ka4x/download

Meteyard, L., & Davies, R. A. (2020). Best practice guidance for linear mixed-effects models in psychological science. *Journal of Memory and Language*, *112*, 104092.

R Core Team. (2018). R: A language and environment for statistical computing. R foundation for statistical computing. Vienna, Austria. R version 3.5.1 (2018-07-02). Retrieved from: http://www.R-project.org
