# Supplementary material for: Semantic priming and schizotypal personality: reassessing the link between thought disorder and enhanced spreading of semantic activation
Source: PeerJ. 2020 Jul 30;8:e9511. doi: 10.7717/peerj.9511 (PMC7396150; doi:10.7717/peerj.9511)
Supplement: Supplemental Information 8 [file peerj-08-9511-s008.pdf]

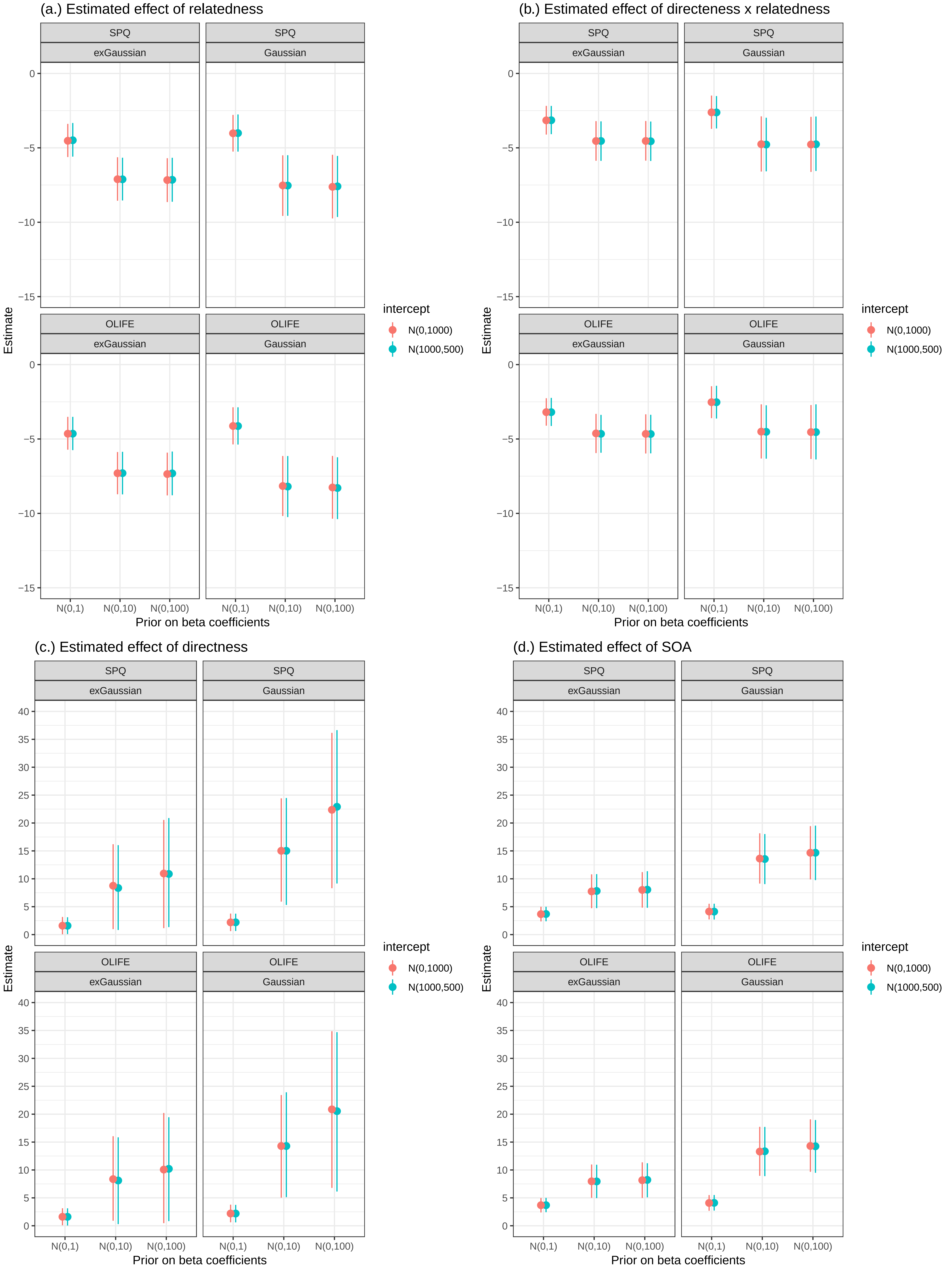

(a.) Estimated effect of relatedness
(b.) Estimated effect of directeness x relatedness
(c.) Estimated effect of directness
(d.) Estimated effect of SOA
SPQ
OLIFE
exGaussian
Gaussian
Estimate
Prior on beta coefficients
N(0,1)
N(0,10)
N(0,100)
intercept
N(0,1000)
N(1000,500)
